# Generalization of Deep Learning in Digital Pathology: Experience in Breast Cancer Metastasis Detection

**DOI:** 10.3390/cancers14215424

**Published:** 2022-11-03

**Authors:** Sofia Jarkman, Micael Karlberg, Milda Pocevičiūtė, Anna Bodén, Péter Bándi, Geert Litjens, Claes Lundström, Darren Treanor, Jeroen van der Laak

**Affiliations:** 1 Department of Clinical Pathology, and Department of Biomedical and Clinical Sciences, Linköping University, 581 83 Linköping, Sweden; 2 Center for Medical Image Science and Visualization (CMIV), Linköping University, 581 85 Linköping, Sweden; 3 Department of Pathology, Radboud University Medical Center, P.O. Box 9101, 6500 HB Nijmegen, The Netherlands; 4 Sectra AB, Teknikringen 20, 583 30 Linköping, Sweden; 5 Leeds Teaching Hospitals NHS Trust, St James´s University Hospital, Beckett Street, Leeds LS9 7TF, UK; 6 Department of Pathology, University of Leeds, Woodhouse Lane, Leeds LS2 9JT, UK

**Keywords:** digital pathology, artificial intelligence, computational pathology, deep learning, generalization, lymph node metastases, breast cancer

## Abstract

**Simple Summary:**

Pathology is a cornerstone in cancer diagnostics, and digital pathology and artificial intelligence-driven image analysis could potentially save time and enhance diagnostic accuracy. For clinical implementation of artificial intelligence, a major question is whether the computer models maintain high performance when applied to new settings. We tested the generalizability of a highly accurate deep learning model for breast cancer metastasis detection in sentinel lymph nodes from, firstly, unseen sentinel node data and, secondly, data with a small change in surgical indication, in this case lymph nodes from axillary dissections. Model performance dropped in both settings, particularly on axillary dissection nodes. Retraining of the model was needed to mitigate the performance drop. The study highlights the generalization challenge of clinical implementation of AI models, and the possibility that retraining might be necessary.

**Abstract:**

Poor generalizability is a major barrier to clinical implementation of artificial intelligence in digital pathology. The aim of this study was to test the generalizability of a pretrained deep learning model to a new diagnostic setting and to a small change in surgical indication. A deep learning model for breast cancer metastases detection in sentinel lymph nodes, trained on CAMELYON multicenter data, was used as a base model, and achieved an AUC of 0.969 (95% CI 0.926–0.998) and FROC of 0.838 (95% CI 0.757–0.913) on CAMELYON16 test data. On local sentinel node data, the base model performance dropped to AUC 0.929 (95% CI 0.800–0.998) and FROC 0.744 (95% CI 0.566–0.912). On data with a change in surgical indication (axillary dissections) the base model performance indicated an even larger drop with a FROC of 0.503 (95%CI 0.201–0.911). The model was retrained with addition of local data, resulting in about a 4% increase for both AUC and FROC for sentinel nodes, and an increase of 11% in AUC and 49% in FROC for axillary nodes. Pathologist qualitative evaluation of the retrained model´s output showed no missed positive slides. False positives, false negatives and one previously undetected micro-metastasis were observed. The study highlights the generalization challenge even when using a multicenter trained model, and that a small change in indication can considerably impact the model´s performance.

## 1. Introduction

Histopathology is a cornerstone in cancer care and is essential for diagnosing, grading, and staging of tumors. Today, pathology services are challenged by a growing shortage of pathologists while at the same time the number and complexity of cases is increasing [1,2,3]. New aids are urgently needed to support pathologists in their diagnostic work, enabling benefits for the cancer patient with shorter time to diagnosis and better targeted treatment. In the last five years, histopathology is increasingly using digitized tissue slides and computer screens as a replacement for the conventional light microscope. Digitization of tissue sections results in so-called whole-slide images (WSI), which open the door for the application of artificial intelligence (AI) and machine learning techniques. Specifically, convolutional neural networks (CNN; a form of deep learning) have been shown to be able to automate tasks in histopathology at a level comparable to that of experienced pathologists [4]. AI has successfully been used to detect and grade prostate cancer, breast cancer, colorectal cancer, and many other malignancies [5,6,7].

An important diagnostic task is identifying metastases of breast cancer in lymph nodes in the axilla of breast cancer patients. Breast cancer is one of the most common and is the most prevalent cancer globally [8]. The number of lymph nodes with metastases and the size of metastases are important parameters in the globally accepted World Health Organization Tumor-Node-Metastasis (TNM) classification system for tumors, which defines cancer staging and lays the ground for treatment strategy and prognosis [9]. Currently, often the sentinel node procedure is applied for initial surgical lymph node resection. In this procedure, the first lymph node(s) (located in the armpit, also called the axilla) that drain the tumor area are identified by injecting a tracer close to the tumor, to evaluate if the cancer has spread from the breast. In case of positive sentinel nodes, or in case of advanced stages of breast cancer, a different surgical procedure is performed, so called axillary lymph node dissection. When performing an axillary node dissection procedure, the surgeon will remove as many lymph nodes as possible from the axilla. The aim for the pathologist is to detect the malignant cells in the lymph nodes by microscopic inspection of a number of tissue sections per lymph node and classify the lymph nodes as positive or negative [9,10,11]. This is a time-consuming and perception-wise challenging task, as potentially small metastases may be overlooked in the large area of normal tissue.

It was previously shown that deep learning can be used to automatically detect metastases in lymph nodes, potentially saving pathologists’ time and increase diagnostic accuracy. The results from the CAMELYON16 challenge show that deep learning models achieve performance comparable with a pathologist interpreting slides, even if the pathologist is unconstrainted in time [12,13]. Moreover, it was shown that pathologists aided by AI possessed increased accuracy while reading time was reduced [14].

However, a remaining challenge that impedes clinical implementation of AI is that AI models can be brittle and drop in performance when applied to new unseen data. The models need to be robust and maintain high performance when introduced to data from new settings and variations to be able to add clinical value [15].

### Aim

The aim of this study was to simulate the first step of clinical implementation of an already multicenter pretrained deep learning model. The model was developed by the computational pathology group at Radboud University Medical Center (UMC), Nijmegen, the Netherlands, and was developed to detect areas of breast cancer metastases in sentinel lymph nodes [16]. In this study, we assessed the generalization potential of the existing model in two steps: first, the pretrained model was tested with new, unseen sentinel node data in a new clinical setting. Secondly, the model’s generalization potential was studied for a different but similar indication. In this case the model was trained on sentinel lymph nodes and tested on sections from axillary lymph node dissections. We decided to exclude slides with findings of only isolated tumor cells (ITC) because the model was not trained for detecting ITC, and the clinical implications of the presence of only ITC is debated. The study highlights the generalization challenge in computational pathology image analysis, both for slides with the same type of tissue but also for slides with a small change in surgical indication. Our results showed that such a change can have a large impact on the model´s performance. Still, with a model that was trained on data from multiple centers and with applied extensive data augmentation, retraining was needed. Further studies are required to explore strategies to overcome the generalization challenge and evaluate which model performance is needed to achieve clinical value.

## 2. Materials and Methods

### 2.1. Datasets

WSI used in this study were collected from two sources. The “AIDA Axillary lymph nodes in breast cancer cases” dataset (AIDA BRLN) from Linköping, Sweden, was used as a local data source. The other source was the CAMELYON datasets from Nijmegen, the Netherlands, that was originally used to train the model that was evaluated in this study. Both datasets are described in more detail below. Negative and positive slides were included. No slides containing only isolated tumor cells were included.

#### 2.1.1. Local Linköping Data

The AIDA BRLN dataset is published at the AIDA Dataset Register and consists of 396 full lymph node cases, totaling 4462 digitized histopathological slides [17]. All lymph nodes were removed from the axilla of breast cancer patients that were admitted, processed, and scanned for clinical use at the Department of Clinical Pathology at Linköping University Hospital, Sweden. AIDA BRLN contains slides from both sentinel node and axillary dissection procedures. The primary tumor is either invasive carcinoma of no special type (NST, also known as invasive ductal carcinoma) or invasive lobular carcinoma (ILC). All slides were stained with hematoxylin-eosin (H&E) and sentinel node cases also include cytokeratin immunohistochemical stained slides (AE1/AE3, Agilent Technologies, Santa Clara, CA, USA). Slides were scanned at a resolution of 0.46 or 0.5 microns per pixel (a small number of slides scanned with a higher magnification). The scanners used were Aperio ScanScope AT (Leica Biosystems, Wetzlar, Germany), Hamamatsu NanoZoomer XR, Hamamatsu NanoZoomer S360 and Hamamatsu NanoZoomer S60 (all Hamamatsu Photonics, Hamamatsu city, Japan). All WSI in AIDA BRLN were anonymized and exported to a research picture archiving and communicating system (PACS) (IDS7, Sectra AB, Linköping, Sweden). 

**Figure 1:**
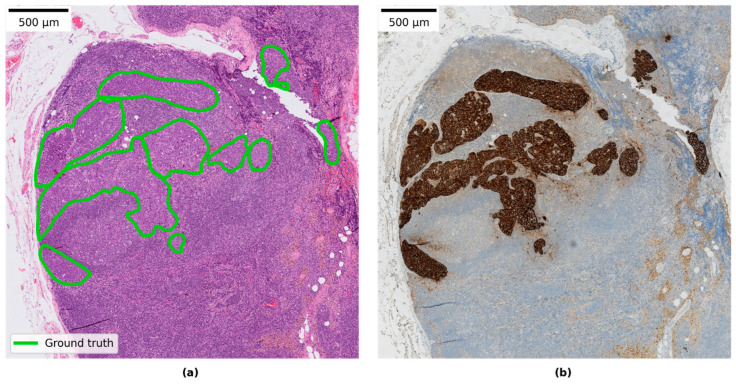
(**a**) Example of annotation of metastasis on an H&E slide. (**b**) Example of corresponding immunohistochemical stained slide for cytokeratin (CK AE1/AE3).

Three subsets of the available slides in AIDA BRLN were defined and used in this study and are referred to as LocalSentinel, LocalAxillary, and LocalNegativeAxillary. Further description of each dataset is listed in Table 1. Ground-truth detailed pixel annotations were produced for the positive slides by a pathology resident (S.J., 4–5 years in practice) by manually delineating separate clusters of tumor cells through visual inspection in the research PACS. When available, cytokeratin immunohistochemical (AE1/AE3) stained slides were used to aid in producing the annotations, an example is shown in Figure 1. All collected slides from the AIDA BRLN dataset were also assigned a ground-truth slide-label as either negative, micro-metastasis or macro-metastasis by a pathologist (S.J.) based on the TNM classification listed in Table 2. A second round for reviewing the slide labels was performed by another pathologist specialized in breast pathology (A.B.), and a consensus diagnosis was discussed in the case of uncertain slides.

#### 2.1.2. CAMELYON Data

CAMELYON16 and CAMELYON17 consist of WSI of both positive and negative lymph node slides from sentinel node procedures and are available on the grand-challenge platform [12]. Included primary tumors in CAMELYON16 are NST and ILC. No information regarding primary tumor subtypes was available for CAMELYON17. H&E slides were scanned at 0.23–0.25 microns per pixel, using a 3DHistech Panoramic Flash II 250 scanner (3DHistech, Budapest, Hungary), a Hamamatsu NanoZoomer-XR C12000-01 scanner (Hamamatsu, Hamamatsu city, Japan), and a Philips Ultrafast Scanner (Philips, Best, Netherlands). See Table 1 for further information on the CAMELYON16 and CAMELYON17 datasets.

#### 2.1.3. Data Splits

All datasets in this study, except LocalNegativeAxillary, were split into non-overlapping training, validation, and test data subsets for which details are listed in Table 3. For CAMELYON16, pre-generated data splits from the CAMELYON organizers were used. For LocalSentinel and LocalAxillary, a stratified random split was used to ensure that a similar ratio of positive to negative slides were present in all subsets. In addition, nine extra negative axillary slides containing only extra-nodal tissue were manually added to the training and validation sets in LocalAxillary to enrich data variation (contained fat necrosis and foreign body reactions). In total, 961 slides were used in this study for model training and evaluation and an additional 259 slides for model evaluation on negative slides (LocalNegativeAxillary).

### 2.2. Study Design

In this study, a state-of-the-art pretrained network from Radboud UMC, Nijmegen, Netherlands was evaluated [16]. This network will be referred to as the base model throughout this study and was trained on CAMELYON data (data from five centers). The base model is a modified classification network of the DenseNet architecture, first published by Huang et al [18]. Please see the "Model architecture” section below for more details on the network.

This study was designed to evaluate the base model’s generalization performance to new data. This evaluation was two-fold. Firstly, the LocalSentinel test set was used to evaluate the generalization performance on same type of data that the model was trained on, i.e., sentinel node data but from another clinical site. Secondly, the model’s generalization performance was evaluated with a different (but similar) type of dataset, in this case with lymph nodes from the same patient group and location (arm-pit) but with another surgical indication and procedure, so-called axillary dissections, using the LocalAxillary test set. The generalization performance of the base model was evaluated with receiver operating curve–area under curve (ROC-AUC) and free-response operating characteristic (FROC), which are described in more detail below. In addition, the potential impacts of primary tumor subtypes were also evaluated. Furthermore, a quantitative evaluation of the base model was performed on 24 negative axillary cases (LocalNegativeAxillary). This enabled us to explore the number of false positives generated in a negative dataset when applying the model to out-of-distribution data, such as a new surgical indication.

Furthermore, the base model was also retrained by pooling the CAMELYON and local data to allow the model to learn new unseen features not present in the CAMELYON datasets alone. Thereby, aiming to make the model more robust to larger data variations related to different clinical settings and indications. The retraining procedure is explained in more detail below. The resulting model will be referred to as the local model henceforth in this paper. A second round of evaluation was then performed to explore the local model´s generalization performance in the same way as described above for the base model.

Concurrently to the generalization evaluation, a qualitative review of the predictions of the local model was performed by a pathologist (S.J.) by visually inspecting model predicted annotations from LocalSentinel and LocalAxillary test sets (68 slides). This allowed us to explore commonalities in tissue morphology that potentially generate false detections by the model, and also to explore how model predictions can be reviewed in a clinical setting. The analysis of the false positives was primarily performed by one pathologist (S.J.) while a secondary review was performed by a second pathologist (D.T.).

### 2.3. Evaluation of WSI Predictions

Model predictions were produced by applying the models in a fully convolutional matter, thus generating prediction score maps (also known as heatmaps) for each WSI, highlighting cancerous regions. The heatmap values range from zero to one where a value of one indicates a high probability of tumor. Furthermore, the resulting heatmaps were used to generate detection regions needed for calculating AUC and FROC by following the outlined procedure in the study from Bejnordi et al. [13]. All detections with diameter less than 0.2 mm were excluded from the evaluations, as these most probably indicate isolated tumor cells (ITCs) which are out of scope for this study. 

Resulting detection regions were converted into coordinate annotations, similar to the drawn ground-truth annotations, using the automated slide analysis platform (ASAP, version 1.9 [19] and uploaded to the research PACS for qualitative review. Finally, a slide label was automatically assigned based on the diameter of the largest detection region which was compared to the TNM classification criteria (see Table 2).

### 2.4. Metrics

Slide-level AUC and lesion-level FROC were used to statistically evaluate all networks in this study. AUC expresses discriminative power between classes healthy and tumor at the slide level [20]. The AUC score was calculated by extracting the pixel value with the highest prediction score from each heatmap, excluding prediction regions smaller than 0.2 mm, and paired with the corresponding ground-truth label. The AUC score ranges from zero to one, where a value of one indicates perfect discrimination between classes. 

For FROC analysis, we evaluated each detection point returned by the algorithm with respect to the annotated ground truth [21]. Points outside any annotation region were considered false positives, whereas points within an annotation are true positives. Annotations that were not assigned any detection points by the algorithm were considered false negatives. The FROC curve, as defined in the CAMELYON challenge, shows the lesion-level, true-positive fraction (sensitivity) relative to the average number of false-positive detections in metastasis-free slides. Furthermore, a single FROC score was defined as the average sensitivity across 6 predefined false-positive rates: 1/4 (1 false-positive result in every 4 WSIs), 1/2, 1, 2, 4, and 8 false-positive findings per WSI. The FROC value ranges from zero to one, where a value of one indicates perfect lesion detection. 

Confidence intervals of 95% for both AUC and FROC were constructed based on 10,000 bootstrap samples of the datasets. Statistical significance was determined with permutation testing using both AUC and FROC as test statistics with 10,000 permutations and a *p*-value of 0.05 [22].

### 2.5. Model Architecture

In this study, a derivative of the DenseNet classification architecture, first developed by Huang et al [18] and later modified by Bándi et al. [16] was used in all experiments. The DenseNet architecture was chosen as it holds state-of-art performance in the CAMELYON17 challenge [23]. For the purpose of this study, it is unlikely that other architectures trained using the same data would show significantly improved generalizability.

More specifically, the modified DenseNet architecture uses valid-padding instead of zero-padding and is composed of 3 dense blocks, each consisting of four 1 × 1 convolutional layers and three 3 × 3 convolutional layers in an alternating manner. Each dense block was followed by a transition block consisting of one 1 × 1 convolutional layer and one 2 × 2 average pooling layer except for the last dense block, which was followed by one 3 × 3 convolutional layer. In the dense blocks, each 1 × 1 convolutional layer had 64 channels whereas each 3 × 3 convolutional layer 32 channels. Additionally, skip connections for layer outputs were added in the dense blocks prior to each 1 × 1 convolution layer and were concatenated downstream with individual outputs from all 3 × 3 to 1 × 1 convolutional layer combinations inside of the dense block. Batch normalization [24] and ReLU non-linearity transformation was applied after each convolution except for the last 3×3 convolutional layer on which a soft-max function was applied instead. 

### 2.6. Model Retraining

All networks in this study were initialized with the He [25] initialization method and the weights were updated using the Adam optimizer [26]. Categorical cross-entropy was used as the loss function together with L2 regularization with a weight of λ = 10^−4^. A pixel spacing of 0.5 micrometers per pixel was used to read the WSIs and a patch size of 279 × 279 pixels was used as input to the network. 262,144 patches were randomly extracted from the WSIs in each epoch for both the training set and validation sets individually. The ratio between tumor and healthy patches was 20/80. A learning rate of *l =* 1 × 10^−4^ was used in the beginning of network training and was reduced by a factor of 10 if no improvement in validation accuracy was observed for 4 consecutive epochs. The models were trained for 200 epochs where early stopping was used if no improvement in validation accuracy were observed for 20 consecutive epochs.

This study made use of several augmentation techniques to further enrich the training dataset. The following augmentations with their corresponding parameter values listed in square brackets were used in the following order, horizontal flipping, rotation [0°, 90°, 180°, and 270°], scaling [0.9, 1.1], hue adjustment [−0.1, 0.1], saturation adjustment [−0.25, 0.25], brightness adjustment [−0.25, 0.25], contrast adjustment [−0.25, 0.25], Gaussian noise [0.0, 0.05], and Gaussian blur [0.0, 1.0]. For additional information regarding the model architecture and the training setup used in this study, please see Bándi et al. [16]. 

Several new retraining strategies were implemented in this study in an attempt to further build on the domain knowledge acquired from the Bándi et al. This to further account for systematic differences between the datasets related to different clinical settings and surgical indications. In summary, three different strategies were implemented in this study to address potential differences between data sources:

1. **Transfer learning:** Two modes were used when retraining the model:
  - The model was retrained from scratch on available data (CAMELYON + local data)
  - The base model was used as a starting point and only trained on the local data (LocalSentinel + LocalAxillary).
2. **Sampling rates:** Two modes for patch sampling from WSI were implemented when both CAMELYON and local data were used:
  - Patches were sampled uniformly from all available images.
  - The patch sampling rate from LocalSentinel and LocalAxillary was increased to allow the model to learn more from the local data and combat fitting preference towards the CAMELYON data which contained the most slides.
3. **Hard negative mining.** Increased sampling rate in negative regions that were often classified as false positives in LocalSentinel and LocalAxillary. See further description of this method below.

Several permutations of the above strategies were implemented and evaluated in this study, see Table S1 in the Supplementary Materials for more information on each implemented permutation. All models were trained and validated on the defined training sets and validation sets seen in Table 3, respectively. The model with the highest performance across all datasets was selected, and its generalization performance was evaluated on the defined test sets.

### 2.7. Hard Negative Mining

**Figure 2:**
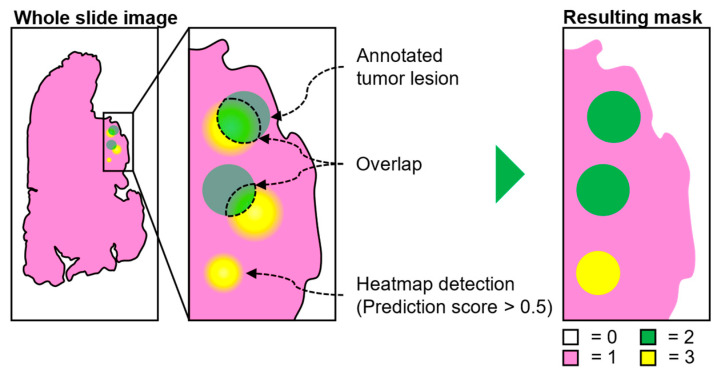
Hard negative mining protocol. Mask generation is based on model generated heatmap (yellow regions) and existing ground-truth annotations (green regions). Heatmap regions overlapping with the ground truth are excluded in the final mask where background (white) is coded as 0, healthy tissue (pink) as 1, ground-truth annotations as 2 (green), and hard negative regions as 3 (yellow).

A simple protocol for adding hard negative regions (i.e., regions which contain tissue morphology that can be mistaken as tumor) to label masks is illustrated in Figure 2 below [27]. The label masks contain spatial information of locations where healthy and tumor regions are located within the slides. An initial model was trained on the full dataset (CAMELYON + local data) and was used to produce whole-slide heatmaps where regions with a prediction score equal to or larger than 0.5 of being tumor were retained (yellow regions). Resulting regions larger than ITCs (>0.2 mm) were added to the label mask if, and only if, no overlap occurred between the predicted region and the ground-truth tumor lesions (green regions).

## 3. Results

### 3.1. Test of Generalisation to a New Setting: Base Model on Local Sentinel Node Data

The base model achieved an AUC of 0.969 (95% CI 0.926–0.998) and FROC of 0.838 (95% CI 0.757–0.913) on CAMELYON16 test data (see Table 4) which is identical to reported values by Bándi et al. [16]. The base model achieved an AUC of 0.929 (95% CI 0.800–0.998) and a FROC of 0.744 (95% CI 0.556–0.912) on LocalSentinel test set, indicating a drop in performance when tested on local data. No statistical difference between the CAMELYON16 and LocalSentinel test sets was observed for either AUC (*p* > 0.05) or FROC (*p* > 0.05).

In addition, the base model was further evaluated on CAMELYON16 and LocalSentinel slides containing only NST as the primary tumor type. All ILC slides were therefore excluded from the CAMELYON16 and LocalSentinel test sets. For CAMELYON16 this resulted in an AUC of 0.976 (95% CI 0.936–0.999) and a FROC of 0.820 (95% CI 0.732–0.902). For LocalSentinel an AUC of 0.992 (95% CI 0.966–1.000) and a FROC of 0.844 (95% CI 0.688–1.000) was observed. No statistical significance was observed between the CAMELYON16 and LocalSentinel test sets in either AUC (*p* > 0.05) or FROC (*p* > 0.05) when ILC slides were excluded.

### 3.2. Test of Generalisation to a New Indication: Base-Model on Local Axillary Dissection Node Data

The base model achieved an AUC of 0.898 (95% CI 0.700–1.000) and a FROC of 0.503 (95% CI 0.201–0.911) on the LocalAxillary test data (see Table 4). The large drop in FROC performance between the CAMELYON16 and LocalAxillary test sets was statistically significant (*p* = 0.012) when using the base model. No statistical significance in model performance was observed between the CAMELYON16 and LocalAxillary test sets (*p* > 0.05) when using AUC as a test statistic.

As before, the base model was further evaluated on LocalAxillary slides containing only NST as the primary tumor type. This resulted in an AUC of 1.000 (95% CI 1.000–1.000) and a FROC of 0.594 (95% CI 0.212–1.000). No statistical significance was observed between the CAMELYON16 and LocalAxillary test sets for AUC (*p* > 0.05) when excluding ILC slides. Statistical significance was observed for FROC (*p* = 0.045) between the CAMELYON16 and LocalAxillary test set when ILC slides were excluded.

Furthermore, 9088 false positive detections were made by the base model on LocalNegativeAxillary (259 negative slides) which corresponds to around 35 detections per slide on average.

### 3.3. CNN Retraining to Increase Performance on Local Data

To mitigate the observed drop in performance, especially for FROC, several new models were trained and validated by including slides from both LocalSentinel and LocalAxillary in the training data. The corresponding performance of all models are reported in Table S2 in the Supplementary Materials. We found that re-training the model on both sentinel and axillary nodes in combination with hard negative mining on axillary lymph nodes resulted in the largest increase in validation performance for both sentinel and axillary data. This model is referred to as the local model henceforth throughout this paper. The local model achieved an AUC of 0.972 (95% CI 0.910–1.000) and a FROC of 0.774 (95% CI 0.592–0.940) for the LocalSentinel test set, and an AUC of 1.000 (95%CI 1.000–1.000) and FROC 0.758 (95% CI 0.632–1.000) for the LocalAxillary test set (see Table 4). No significant change in performance was observed in the LocalSentinel test set between the base model and the local model for either AUC (*p* > 0.05) or FROC (*p* > 0.05). A significant performance increase for AUC was observed in the LocalAxillary test set between models (*p* = 0.019) whereas no significant change in performance was observed for the FROC value in the LocalAxillary test set (*p* > 0.05).

The local model was further evaluated on the datasets in which ILC had been excluded. This resulted in an AUC of 0.997 (95% CI 0.985–1.000) and a FROC of 0.870 (95% CI 0.706–1.000) for the LocalSentinel test set, and an AUC of 1.000 (95%CI 1.000–1.000) and FROC 0.744 (95% CI 0.619–1.000) for the LocalAxillary test set (see Table 4). No significant performance change was observed between models in the LocalSentinel test set with excluded ILC slides for either AUC (*p* > 0.05) or FROC (*p* > 0.05). Similarly, no significant change was observed between models in the LocalAxillary test set with excluded ILC slides for either AUC (*p* > 0.05) or FROC (*p* > 0.05).

In addition, 327 false positive detections were made by the local model on LocalNegativeAxillary which is a little more than 27 times fewer compared to the base model. On average, there were about 1.3 false positive detections per slide.

### 3.4. Qualitative Evaluation of Local Model Predictions

**Figure 3:**
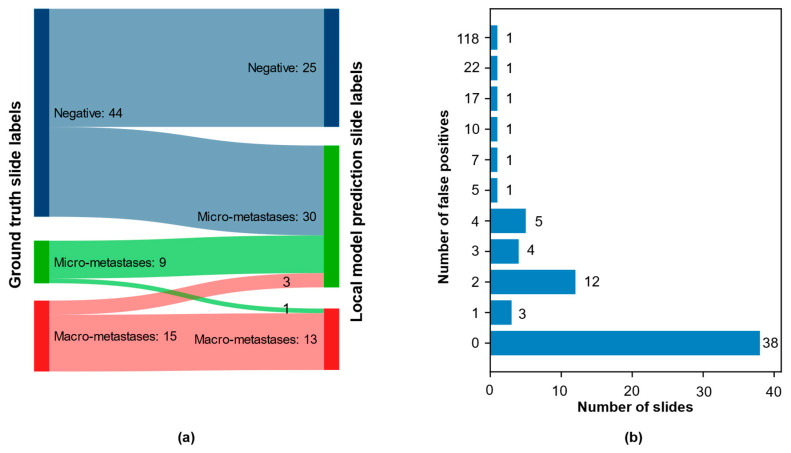
Results from pathologist qualitative evaluation of local model predictions: (**a**) Sankey diagram over the combined test set (LocalSentinel + LocalAxillary), n = 68, with the ground-truth slide label on left side and local model prediction slide label on right side. Numbers represent the number of slides in each diagnosis group. (One negative slide in ground truth misclassified as negative); and (**b**) the distribution of false positive across the 68 slides: 38 slides had no false positive; most of the slides with false positives contained 1-4 false positive; a small number of slides had a large number of false positives.

**Figure 4:**
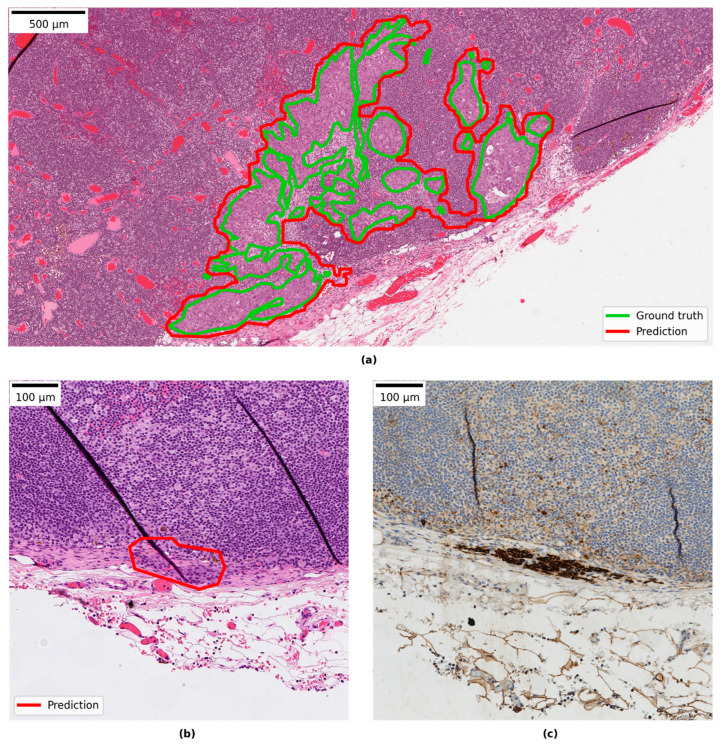
(**a**) Example of ground-truth annotation and model prediction detection area overlapping; (**b**) example of correct predicted micro-metastasis missed by ground truth; and (**c**) the corresponding immunohistochemistry slide with cytokeratin positive cells.

The combined test set from LocalSentinel and LocalAxillary consisted of 68 slides. The ground-truth slide labels and the local model slide labels matched in 45 of the 68 (66%) slides. Among the positive slides, the slide label matched in 20 of 24 slides (83%). No positive slides in ground truth were wrongly assigned as negative by the model. See Figure 3a for more details. For example of ground truth annotation and model prediction detection area overlapping see Figure 4a. In one slide the model detected a micro-metastasis, that was missed in the ground-truth generation. The micro-metastasis was confirmed in the immunohistochemical stained slide (see Figure 4b,c) and was reviewed by three pathologists (S.J., A.B., D.T.). That slide was also reported as negative in the clinical system. In the remaining negative slides, the slide labels matched in 25 of 43 slides (58%). All mislabeled negative slides were assigned as micro-metastasis by the model.

**Figure 5:**
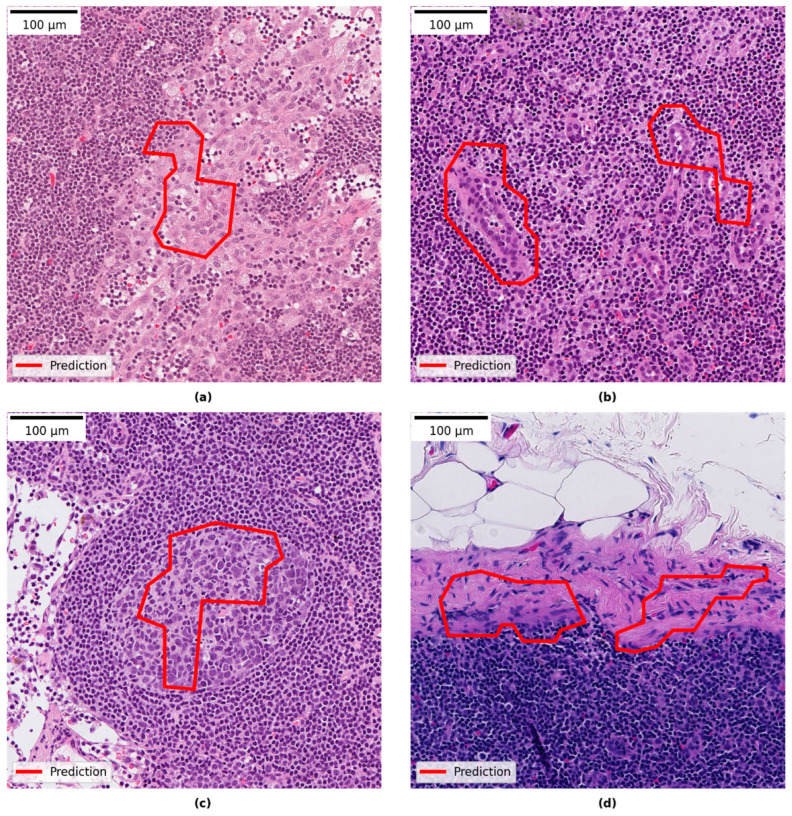
Examples of false positive areas: (**a**) histiocytes; (**b**) vascular elements; (**c**) germinal center; and (**d**) capsular region.

Reviewing the slides in the research PACS showed that 30 of the 68 slides had areas predicted by the model which were false positive. Review of those false positive areas showed that they comprised benign pathological features. The most common category was histocytes, then in reducing order of frequency: vascular elements, germinal centers, capsular region and fibrotic elements, and subcapsular region. The smallest category was other features such as artefactually damaged tissue, eosinophilic amorphous material and plasma cells. Most of the slides with false positives contained between 1–4 false positive areas (see Figure 3b). For examples of false positive areas see Figure 5. The model also showed false negative areas. In five slides there were false negative areas corresponding to the size of micro-metastases, and the number of areas per slide ranged between 1–7. In no cases did that change the slide label diagnosis. In two slides large areas of missed malignant cells inside larger metastases were observed.

## 4. Discussion

Today, there is a gap between deep learning models showing good (i.e., human-level) performance in experimental evaluation and examples of successful clinical implementations. Reasons for this could be a lack of digitization among pathology laboratories, pathologists not trusting the computer models, and regulatory matters. But one of the greatest challenges is the generalization of the models, i.e., its ability to maintain high accuracy on novel data or situations. The models must be robust and maintain high performance on slides from different laboratories if AI is to be successful in clinical practice. In a pathology laboratory there are many potential sources of data variations. For example, many of the laboratory steps, such as differences in staining protocol, tissue quality, and thickness of the sections could render color variation. Gray et al. showed, for example, that slides stained on different days varied considerably compared to slides stained on the same day based on experiments using image analysis color deconvolution. They could also show staining variability between automated staining instruments, between regional laboratories and between scanners [28]. In training state-of-the-art AI models, a large amount of data is needed from several centers to cover data variation and to prevent the model from becoming overfitted to training data [29]. Obtaining well-curated and annotated data, however, is a substantial bottleneck. The training of models in computational pathology is often based on supervised learning, which requires detailed annotations made by expert pathologists. This is a slow and laborious process, limiting the amount of available high-quality data. To mitigate potentially too small dataset sizes, different pre-processing steps can be used such as image augmentations which was used in this study. Image augmentation is a technique to introduce variation of the data in the already existing dataset in an attempt to overcome the challenge of obtaining large hand-curated datasets. Extracted patches used for training are altered, e.g., flipping the image horizontally or vertically, rotating the image, introducing random noise, and change in color intensities to mention a few [30,31].

In this study, we evaluated the generalizability performance of a multi-center pretrained model with previously state-of-the-art performance in detecting breast cancer metastases in lymph nodes of sentinel node type. When we tested the base model on unseen local sentinel node data a performance drop was observed. The performance drop however was not seen when only NST slides were evaluated (excluding ILC slides). This is an indication that the model struggles to correctly classify the ILC slides. In our data test sets (LocalSentinel and LocalAxillary), the amount of ILC slides is more than twice (~33%) of that present in CAMELYON16 (~12%), thus the impact on AUC and FROC will be larger in LocalSentinel and LocalAxillary when ILC slides are incorrectly classified. NST and ILC are the two most common subtypes in breast cancer and differ in morphology. Tang et al. describe in an example that the risk of overfitting a model with limited data for a heterogeneous cancer type can be detrimental to the generalization performance and demonstrating that adding additional subtypes of osteosarcoma increases the model’s performance and robustness [32].

Other sources of variation between the data could be differences in scanning manufacturers and magnification, or surgical method of tracing the sentinel lymph node. Another aspect could also be that the local data was retroactively collected directly from a clinical digital archive where all slides were scanned in a large-scale scanning process with minor manual interference and will represent the whole clinical spectrum of slide quality, scanning artifacts and special features such as necrotic lymph nodes and extra-glandular tumor deposits.

Secondly, we wanted to test the generalization potential of the multicenter-trained model to another but similar surgical indication. The base model, trained on sentinel nodes, was tested on local lymph nodes from axillary dissections, with a considerable drop in performance, especially in FROC. The performance difference in FROC between CAMELYON16 and LocalAxillary was still pronounced even when ILC was excluded. The result indicates that a small difference in surgical indication, i.e., in this case the lymph nodes originate from the same patient group (breast cancer) and same topography (axilla), can have a large impact on model performance. In qualitative analysis of the model’s predictions on axillary lymph nodes in the PACS we could see that the base model rendered a large number of false positives with the same pattern as false positives in sentinel nodes but with additional features i.e., fat necrosis and foreign body reaction. Those areas are presumably more apparent in axillary nodes while those nodes often have been preceded by another intervention, such as sentinel node procedure, as they are reactive changes seen post-operatively.

Our results showed that retraining was needed, especially in the case of axillary lymph nodes to mitigate the performance drop. The most successful retraining strategy was a combination of training of patches sampled uniformly from all slides and hard negative mining on axillary lymph nodes resulting in improved performance for both sentinel nodes and axillary nodes. With the retrained model the AUC results on local data are comparable with AUC results with the CAMELYON16 data. The FROC for local data is still inferior to FROC for CAMELYON16, but the result indicates that a large increase was gained for axillary nodes after retraining.

The retrained model shows a high AUC, meaning a good performance in differentiating negative from positive slides. However, important to note is that AUC in this study is calculated by extracting the pixel with the highest prediction score in each slide, meaning if there is a good separation of score values between negative and positive slides, a high AUC value will be obtained. Furthermore, in cases where an AUC of 1.0 is observed, there exists a threshold which allows for perfect class separation. However, due to using the pixel with the highest prediction score from each slide to represent the slide labels (positive or negative), an optimistic performance bias is introduced that only holds true for the slide-level predictions. On the other hand, lesion-level predictions can only be evaluated by using FROC for which lesion-prediction scores will be more varied and, in many cases, lower than the pixel with the highest prediction score in the slide. Caution should therefore be observed to not evaluate the model solely with AUC, but should instead be combined with the FROC value and a qualitative review. This to find a good balance between retaining a high slide-level accuracy and limiting the number of false positive detections in the individual slides. 

In the study 44% of the test slides contained a false positive area, and in the dataset with only negative slides from axillary nodes, there were an average of 1.3 false positives per slide. Most of the false positives represented well-known morphologies of non-tumorous features, some known to be difficult for AI models such as germinal centers and histiocytes [14] but can often easily be rejected by the pathologist. Some amount of false positives will probably be accepted as a reasonable “cost” for not missing malignant areas, but if too many false positives are flagged then user trust in the AI may be lost. Different ways of thresholding the model’s output can be useful for decreasing the number of false positives or avoiding false negative predictions. A comprehensive list of popular post-processing steps in digital pathology was recently reviewed by Salvi et al. [33]. Lindvall et al. and Steiner et al. describes clinical approaches of AI models and how to mitigate imperfect AI and false positives distracting the pathologists by limiting the number of regions displayed for pathologists, but still maintain quality of diagnostics and even reduced review time. Both papers from Lindvall et al. and Steiner et al. show promising results of a synergistic effect of the combination of pathologist and AI-model [14,34].

In the qualitative evaluation of the model predictions, we saw that no positive slides were missed by the model. This could lay ground for a clinical approach of an aiding tool triaging the positive cases first in order to be diagnosed by the pathologist, to shorten lead time to a malignant diagnosis. The retrained model correctly assigned slide-label diagnosis in 20 of 24 positive slides and mismatched micro- and macro-metastasis in four of the positive slides. A possible model of assistive AI deployment could be to let the model outline metastases, but when measuring the metastases, the pathologist could take other factors into account such as measuring two areas together that belong to same tumor.

Regarding limitations in this study the exclusion of ITC can be noted. The model was not trained to detect ITC and no slides with only ITC were included. ITC is reported clinically if detected but still the sentinel lymph nodes are considered as negative according to the TNM-classification, and the clinical value of ITC, or even micro-metastasis, in sentinel node is debatable [35]. However, when examining sentinel node after preoperative treatment any size of viable rest of metastasis is relevant [11,36]. Furthermore, of concern could be the missed malignant areas by the local model, although it did not change slide-level diagnosis. We think further studies are needed to evaluate the impact of those missed malignant areas, for example in a clinical reader-study where multiple full cases are used.

Future studies are required to overcome the challenges of generalizability. Large, multi-center datasets are of paramount importance to train robust deep learning models. This ideally includes different surgical protocols and a good representation of breast cancer subtypes. Large scale initiatives, in which multicentric data is collected and made available to the community at large, may play a major role in the future. One such example is the Bigpicture project, that aims to collect WSI from all of Europe [37].

To reduce data dependence, strategies for model training without the need for detailed (pixel-level) ground-truth annotations could potentially accelerate the development. To better appreciate the impact that AI can have on pathology diagnostics, and also understand the performance requirements of the AI models, further studies are needed in which AI is validated in a real-world clinical setting. Today, little is known on what exact model performance is needed to generate clinical value. AI models will not stand alone but will rather work as an aid to the pathologist, in which the pathologist will decide on the diagnosis. The next steps in our research will be to explore user interface design, in combination with model performance, to assess potential added clinical value for histopathology cancer diagnostics. 

## 5. Conclusions

The study highlights the generalization challenge in computational pathology where we tested a multicenter pretrained deep learning model on unseen lymph node data of the same type of tissue it was trained for and also introduced data with a small change in surgical indication, i.e., lymph nodes from axillary dissections. Retraining with targeted data was needed in both scenarios to mitigate the drop in performance. The result especially highlights that a small change in indication can impact the model’s performance considerably. The retrained model showed no missed positive slides, but in a few of those slides, areas of malignant cells corresponding to the size of a micro-metastasis were missed. The study highlights the need for further work on strategies overcoming the generalization challenge and the need for evaluation of what model performance is needed to add clinical value.

## Figures and Tables

**Table 1:** Description of datasets used in the study.

Dataset	Unique Cases	Slides	Description
LocalSentinel	161	161	Retrospectively collected lymph node slides from sentinel node procedures consisting of 107 negative and 54 positive slides. One slide per unique patient case included. Cytokeratin immunohistochemically stained sections were available (AE1/AE3) and were used to aid in production of detailed ground-truth annotations. (Subset of AIDA BRLN dataset.)
LocalAxillary	48	57	Retrospectively collected lymph node slides from axillary dissection procedures consisting of 24 negative and 24 positive slides from unique cases. This dataset was further enriched with nine extra negative slides, overlapping with other cases, which contained only extra-nodal tissue and featured examples of fat necrosis and foreign body tissue reactions. (Subset of AIDA BRLN dataset.)
LocalNegativeAxillary	24	259	Retrospectively collected lymph node cases from axillary dissection procedures that were signed out as negative during initial clinical diagnostic assessment. All slides in the cases were included except for slides (n = 33) already included in LocalSentinel or LocalAxillary to avoid overlapping slides. One section per block, except in four cases 1–3 additional sections per block were presented in the clinical archive and included in this set. (Subset of AIDA BRLN dataset.)
CAMELYON16	399	399	Retrospectively collected sentinel lymph node slides from two hospitals in the Netherlands. Pre-generated data splits by the CAMELYON organizers were retained in this study. Of the 160 positive slides, detailed annotations were available for 140 slides, whereas 20 slides were only partially annotated e.g., slides that contained two consecutive sections of the same tissue or slides that contained out-of-focus tumor regions, and only the delineated regions containing tumors were used in these slides during training.
CAMELYON17	200	1000 (344 used)	Retrospectively collected sentinel lymph node slides from five hospitals in the Netherlands. A total of 1000 slides. Only a subset of 50 positive slides contained detailed annotations, while the remaining positive slides had been given a slide label according to the TNM staging system. Of the available 50 annotated positive slides, only 34 slides contained either micro- or macro-metastases and were included in this study, the remaining 16 slides were labelled as isolated tumor cells (ITC) and were excluded. 50 randomly selected negative slides from the CAMELYON17 training set and 260 negative slides in the CAMELYON17 test set were allocated to training and testing sets, respectively, in this study. In total 344 slides from CAMELYON17 were used in this study.

**Table 2:** Slide-label classification based on the WHO TNM classification system [9].

Type	Description	Slide Label
Macro-metastasis	Tumor > 2 mm	Positive
Micro-metastasis	Tumor > 0.2 mm and ≤ 2 mm	Positive
Isolated tumor cells (ITC)	Tumor ≤ 0.2 mm or ≤ 200 tumor cells	Negative
No tumor cells	No tumor cells	Negative

**Table 3:** Distribution of WSI for training, validation, and test of four of the datasets. Italicized numbers indicate added slides with extra-nodal tissue containing features of fat necrosis and foreign body tissue reaction.

Dataset	Summary			Training			Validation			Testing		
	N	Neg	Pos	N	Neg	Pos	N	Neg	Pos	N	Neg	Pos
CAMELYON16	399	239	160	216	127	89	54	32	22	129	80	49 ^(1)^
CAMELYON17	344	310	34	55	40	15	19	10	9	270	260	10
LocalSentinel	161	107	54	88	58	30	22	15	7	51	34	17 ^(2)^
LocalAxillary	48 + *9*	24 + *9*	24	24 + *6*	11 + *6*	13	7 + *3*	3 + *3*	4	17	10	7 ^(3)^
**Total**	**961**	**689**	**272**	**389**	**242**	**147**	**105**	**63**	**42**	**467**	**384**	**83**

^(1)^ Consists of 6 ILC slides (12%) and 43 NST slides (88%); ^(2)^ Consists of 6 ILC slides (35%) and 11 NST slides (65%); ^(3)^ Consists of 2 ILC slides (28%) and 5 NST slides (72%).

**Table 4:** Results for base model and local model on defined testset with AUC and FROC. Metrics were calculated individually on each dataset. The second and fourth row show the results for datasets containing only NST slides.

	LocalSentinel		LocalAxillary		CAMELYON16		CAMELYON17	
	AUC	FROC	AUC	FROC	AUC	FROC	AUC	FROC
Model	(95% CI)	(95% CI)	(95% CI)	(95% CI)	(95% CI)	(95% CI)	(95% CI)	(95% CI)
Base	0.929	0.744	0.898	0.503	0.969	0.838	0.997	0.967
	(0.800–0.998)	(0.566–0.912)	(0.700–1.000)	(0.201–0.911)	(0.926–0.998)	(0.757–0.913)	(0.990–1.000)	(0.886–1.000)
Base	0.992	0.844	1.000	0.594	0.976	0.820	-	-
(only NST)	(0.966–1.000)	(0.688–1.000)	(1.000–1.000)	(0.212–1.000)	(0.936–0.999)	(0.732–0.902)	-	-
Local	0.972	0.774	1.000	0.758	0.981	0.825	0.976	0.910
	(0.910–1.000)	(0.592–0.940)	(1.000–1.000)	(0.632–1.000)	(0.953–1.000)	(0.746–0.900)	(0.926–1.000)	(0.722–1.000)
Local	0.997	0.870	1.000	0.744	0.988	0.817	-	-
(only NST)	(0.985–1.000)	(0.706–1.000)	(1.000–1.000)	(0.619–1.000)	(0.965–1.000)	(0.730–0.896)	-	-

## Data Availability

The BRLN lymph node set is published at the AIDA Dataset Register at https://datahub.aida.scilifelab.se/10.23698/aida/brln (accessed on 1 September 2021). For data sharing policy see https://datahub.aida.scilifelab.se/sharing/policy/ (accessed on 21 May 2022). The CAMELYON dataset is publicly available at https://camelyon17.grand-challenge.org/Data/ (accessed on 2 December 2021).

## Supplementary Materials

The following supporting information can be downloaded at: https://www.mdpi.com/article/10.3390/cancers14215424/s1, Table S1: Model re-training strategies; Table S2: Summary of whole-slide results on the validation sets.

## References

[1] (2019). Guide for Establishing a Pathology Laboratory in the Context of Cancer Control.

[2] Märkl B., Füzesi L., Huss R., Bauer S., Schaller T. (2021). Number of pathologists in Germany: Comparison with European countries, USA, and Canada. Virchows Arch..

[3] Beckman Suurküla M. (2012). Svensk Patologi—En Översyn och Förslag till Åtgärder; A Review of Clinical Pathology in Sweden Ordered by the Swedish Government. Översynen har Genomförts på Regeringens Uppdrag (S2011/5140/FS); Sweden. https://medlem.foreningssupport.se/foreningar/uploads/L15178/F%C3%B6reningen/Rapport_patologi_12.03.29.pdf.

[4] van der Laak J., Litjens G., Ciompi F. (2021). Deep learning in histopathology: The path to the clinic. Nat. Med..

[5] Ström P., Kartasalo K., Olsson H., Solorzano L., Delahunt B., Berney D.M., Bostwick D.G., Evans A.J., Grignon D.J., Humphrey P.A. (2020). Artificial intelligence for diagnosis and grading of prostate cancer in biopsies: A population-based, diagnostic study. Lancet Oncol..

[6] Acs B., Rantalainen M., Hartman J. (2020). Artificial intelligence as the next step towards precision pathology. J. Intern. Med..

[7] Bulten W., Balkenhol M., Jean-Joël, Belinga A., Brilhante A., Çakır A., Egevad L., Eklund M., Farré X., Geronatsiou K. (2021). Artificial intelligence assistance significantly improves Gleason grading of prostate biopsies by pathologists ISUP Pathology Imagebase Expert Panel. Mod. Pathol..

[8] Breast Cancer. https://www.who.int/news-room/fact-sheets/detail/breast-cancer.

[9] Brierley J.D., Gospodarowicz M.K., Wittekind C. (2017). TNM Classification of Malignant Tumours.

[10] Apple S.K. (2016). Sentinel Lymph Node in Breast Cancer: Review Article from a Pathologist’s Point of View. J. Pathol. Transl. Med..

[11] (2022). Bröstcancer Nationellt Vårdprogram Version: 4.0 (National Program for Breast Cancer in Sweden).

[12] Litjens G., Bandi P., Bejnordi B.E., Geessink O., Balkenhol M., Bult P., Halilovic A., Hermsen M., van de Loo R., Vogels R. (2018). 1399 H&E-stained sentinel lymph node sections of breast cancer patients: The CAMELYON dataset. GigaScience.

[13] Bejnordi B.E., Veta M., Van Diest P.J., Van Ginneken B., Karssemeijer N., Litjens G., Van Der Laak J.A.W.M., Hermsen M., Manson Q.F., Balkenhol M. (2017). Diagnostic assessment of deep learning algorithms for detection of lymph node metastases in women with breast cancer. J. Am. Med. Assoc..

[14] Steiner D.F., Macdonald R., Liu Y., Truszkowski P., Hipp J.D., Gammage C., Thng F., Peng L., Stumpe M.C. (2018). Impact of Deep Learning Assistance on the Histopathologic Review of Lymph Nodes for Metastatic Breast Cancer. Am. J. Surg. Pathol..

[15] Kleppe A., Skrede O.-J., De Raedt S., Liestøl K., Kerr D.J., Danielsen H.E. (2021). Designing deep learning studies in cancer diagnostics. Nat. Rev. Cancer.

[16] Bándi P., Balkenhol M., van Dijk M., van Ginneken B., van der Laak J., Litjens G. (2022). Domain adaptation strategies for cancer-independent detection of lymph node metastases. arXiv.

[17] Jarkman S., Lindvall M., Hedlund J., Treanor D., Lundstrom C., van der Laak J. (2019). Axillary lymph nodes in breast cancer cases. AIDA Data Hub (AIDA Dataset Regist.).

[18] Huang G., Liu Z., Van Der Maaten L., Weinberger K.Q. Densely Connected Convolutional Networks. Proceedings of the 30th IEEE Conference on Computer Vision and Pattern Recognition, CVPR 2017.

[19] ASAP—Fluid Whole-Slide Image Viewer—Diagnostic Image Analysis Group. https://www.diagnijmegen.nl/software/asap/.

[20] Bradley A.P. (1997). The use of the area under the ROC curve in the evaluation of machine learning algorithms. Pattern Recognit..

[21] Chakraborty D.P. (2011). Recent developments in imaging system assessment methodology, FROC analysis and the search model. Nucl. Instrum. Methods Phys. Res. Sect. A Accel. Spectrometers Detect. Assoc. Equip..

[22] Efron B., Tibshirani R.J. (1993). An Introduction to the Bootstrap.

[23] Bándi P., Geessink O., Manson Q., van Dijk M., Balkenhol M., Hermsen M., Ehteshami Bejnordi B., Lee B., Paeng K., Zhong A. (2018). From Detection of Individual Metastases to Classification of Lymph Node Status at the Patient Level: The CAMELYON17 Challenge. IEEE Trans. Med. Imaging.

[24] Ioffe S., Szegedy C. Batch Normalization: Accelerating Deep Network Training by Reducing Internal Covariate Shift. Proceedings of the 32nd International Conference on Machine Learning.

[25] He K., Zhang X., Ren S., Sun J. Delving deep into rectifiers: Surpassing human-level performance on imagenet classification. Proceedings of the International Conference on Computer Vision.

[26] Kingma D.P., Ba J. (2014). Adam: A method for stochastic optimization. arXiv.

[27] Wang D., Khosla A., Gargeya R., Irshad H., Beck A.H. (2016). Deep Learning for Identifying Metastatic Breast Cancer. arXiv.

[28] Gray A., Wright A., Jackson P., Hale M., Treanor D. (2015). Quantification of histochemical stains using whole slide imaging: Development of a method and demonstration of its usefulness in laboratory quality control. J. Clin. Pathol..

[29] Shen D., Wu G., Suk H.I. (2017). Deep Learning in Medical Image Analysis. Annu. Rev. Biomed. Eng..

[30] Shorten C., Khoshgoftaar T.M. (2019). A survey on Image Data Augmentation for Deep Learning. J. Big Data.

[31] Tellez D., Litjens G., Bándi P., Bulten W., Bokhorst J.M., Ciompi F., van der Laak J. (2019). Quantifying the effects of data augmentation and stain color normalization in convolutional neural networks for computational pathology. Med. Image Anal..

[32] Tang H., Sun N., Shen S. (2021). Improving Generalization of Deep Learning Models for Diagnostic Pathology by Increasing Variability in Training Data: Experiments on Osteosarcoma Subtypes. J. Pathol. Inform..

[33] Salvi M., Acharya U.R., Molinari F., Meiburger K.M. (2021). The impact of pre- and post-image processing techniques on deep learning frameworks: A comprehensive review for digital pathology image analysis. Comput. Biol. Med..

[34] Lindvall M., Lundström C., Löwgren J. Rapid Assisted Visual Search Supporting Digital Pathologists with Imperfect AI. Proceedings of the 26th International Conference on Intelligent User Interfaces (IUI ’21).

[35] Houvenaeghel G., de Nonneville A., Cohen M., Chopin N., Coutant C., Reyal F., Mazouni C., Gimbergues P., Azuar A.S., Chauvet M.P. (2021). Lack of prognostic impact of sentinel node micro-metastases in endocrine receptor-positive early breast cancer: Results from a large multicenter cohort☆. ESMO Open.

[36] Viale G., Fusco N. (2022). Pathology after neoadjuvant treatment—How to assess residual disease. Breast.

[37] Moulin P., Grünberg K., Barale-Thomas E., Der Laak J.V. (2021). IMI—Bigpicture: A Central Repository for Digital Pathology. Toxicol. Pathol..

