# Supplementary material for: Generalization of Deep Learning in Digital Pathology: Experience in Breast Cancer Metastasis Detection
Source: Cancers (Basel). 2022 Nov 3;14(21):5424. doi: 10.3390/cancers14215424 (PMC9659028; doi:10.3390/cancers14215424)
Supplement: Supplementary file 1 [file cancers-14-05424-s001.zip › cancers-1903281 - supplementary.pdf]

cancers MDPI

Table S1. Strategies for retraining models. The base model from Radboud University medical center (UMC), the Netherlands, is highlighted in orange. The selected retrained model is highlighted in green and is referred to as local model in the study.

Model	CAMELYON16 (CAM16)	CAMELYON17 (CAM17)	LocalSentinel	LocalAxillary	Training Strategy
**Base model**	X	X			Original model received from Radboud UMC. Based on DenseNet architecture.
**Model 1**	X	X	X	X	Model trained on a pooled dataset (CAM16, CAM17, LocalSentinel, and LocalAxillary data). **Sampling:** 158 images were randomly selected at each epoch with 56% from CAM16, 14% from CAM17, 23% LocalSentinel and 7% from LocalAxillary. A total of 262 144 patches were sampled from the selected images with a positive (tumor) to negative (healthy) patch distribution of 20/80.
**Model 2**	X	X	X	X	Model trained on pooled dataset. **Sampling:** 48 images were randomly selected at each epoch with 33% from CAM16, 25% from CAM17, 25% LocalSentinel and 17% from LocalAxillary. A total of 65 536 patches were sampled from the selected images with a positive to negative patch distribution of 20/80.
**Model 3**			X	X	Transfer learning with echo_alligator as pretrained network and trained on LocalSentinel and LocalAxillary data only. **Sampling:** 158 images were randomly selected at each epoch with 74% LocalSentinel and 26% from LocalAxillary. A total of 262 144 patches were sampled from the selected images with a positive (tumor) to negative (healthy) patch distribution of 20/80.
**Model 4 (Local model)**	X	X	X	X	Pooled datasets with additional hard negative mining of false positives in the **LocalAxillary** dataset. **Sampling:** 158 images were randomly selected at each epoch with 56% from CAM16, 14% from CAM17, 23% LocalSentinel and 7% from LocalAxillary. A total of 262 144 patches were sampled from the selected images with a positive, negative, and hard negative patch distribution of 20/75/5.
**Model 5**	X	X	X	X	Pooled datasets with additional hard negative mining of false positives in the **LocalAxillary** dataset. **Sampling:** 158 images were randomly selected at each epoch with 56% from CAM16, 14% from CAM17, 23% LocalSentinel and 7% from LocalAxillary. A total of 262 144 patches were sampled from the selected images with a positive, negative, and hard negative patch distribution of 20/78/2.
**Model 6**	X	X	X	X	Pooled datasets with additional hard negative mining of false positives in the **LocalAxillary** dataset. **Sampling:** 48 images were randomly selected at each epoch with 33% from CAM16, 25% from CAM17, 25% LocalSentinel and 17% from LocalAxillary. A total of 65 536 patches were sampled from the selected images with a positive, negative, and hard negative patch distribution of 20/75/5.

**Model 7**	X	X	X	X	Pooled datasets with additional hard negative mining of false positives in the **LocalAxillary** dataset.<br>**Sampling:** 48 images were randomly selected at each epoch with 33% from CAM16, 25% from CAM17, 25% LocalSentinel and 17% from LocalAxillary. A total of 65 536 patches were sampled from the selected images with a positive, negative, and hard negative patch distribution of 20/78/2.
**Model 8**	X	X	X	X	Pooled datasets with additional hard negative mining of false positives in **LocalSentinel** and **LocalAxillary** datasets.<br>**Sampling:** 158 images were randomly selected at each epoch with 56% from CAM16, 14% from CAM17, 23% LocalSentinel and 7% from LocalAxillary. A total of 262 144 patches were sampled from the selected images. Hard negative regions in LocalSentinel and LocalAxillary shared same mask label (=3). A positive, negative, and hard negative patch distribution of 20/70/10 was used.
**Model 9**	X	X	X	X	Pooled datasets with additional hard negative mining of false positives in **sentinel** and **axillary** lymph nodes.<br>**Sampling:** 158 images were randomly selected at each epoch with 56% from CAM16, 14% from CAM17, 23% LocalSentinel and 7% from LocalAxillary. A total of 262 144 patches were sampled from the selected images. Individual mask labels for hard negative regions in LocalSentinel (=3) and LocalAxillary (=4) was used. A positive, negative, hard negative (LocalSentinel), and hard negative (LocalAxillary) patch distribution of 20/70/5/5 was used.

Table S2. Summary of whole slide results on the validation sets of LocalSentinel, LocalAxillary, CAM16, and CAM17 using ROC-AUC and FROC. Values in parentheses shows the 95% confidence interval approximated from 10000 bootstrap iterations. The base model from Radboud UMC is highlighted in orange. The selected retrained model is highlighted in green and is referred to as local model in the study.

	**LocalSentinel**		**LocalAxillary**		**CAMELYON16**		**CAMELYON17**	
**Model**	**AUC** *(95% CI)*	**FROC** *(95% CI)*	**AUC** *(95% CI)*	**FROC** *(95% CI)*	**AUC** *(95% CI)*	**FROC** *(95% CI)*	**AUC** *(95% CI)*	**FROC** *(95% CI)*
**Base model**	0.913 *(0.695-1.000)*	0.855 *(0.580-1.000)*	0.584 *(0.167-1.000)*	0.439 *(0.139-0.833)*	0.997 *(0.987-1.000)*	0.977 *(0.977-1.000)*	1.000 *(1.000-1.000)*	0.917 *(0.840-0.992)*
**Model 1**	0.904 *(0.671-1.000)*	0.883 *(0.645-1.000)*	1.000 *(1.000-1.000)*	0.802 *(0.600-1.000)*	1.000 *(1.000-1.000)*	0.981 *(0.951-1.000)*	0.945 *(0.945-1.000)*	0.757 *(0.472-0.976)*
**Model 2**	0.913 *(0.694-1.000)*	0.837 *(0.571-1.000)*	0.875 *(0.500-1.000)*	0.629 *(0.222-0.920)*	0.980 *(0.928-1.000)*	0.969 *(0.912-0.998)*	1.000 *(1.000-1.000)*	0.586 *(0.300-0.905)*
**Model 3**	0.933 *(0.759-1.000)*	0.906 *(0.667-1.000)*	1.000 *(1.000-1.000)*	0.709 *(0.515-1.000)*	0.974 *(0,925-1.000)*	0.967 *(0.919-1.000)*	1.000 *(1.000-1.000)*	0.885 *(0.754-1.000)*
**Model 4 (Local model)**	0.914 *(0.694-1.000)*	0.875 *(0.625-1.000)*	1.000 *(1.000-1.000)*	0.883 *(0.762-1.000)*	1.000 *(1.000-1.000)*	0.982 *(0.946-1.000)*	0.978 *(0.898-1.000)*	0.790 *(0.539-1.000)*
**Model 5**	0.884 *(0.607-1.000)*	0.861 *(0.583-1.000)*	1.000 *(1.000-1.000)*	0.849 *(0.741-1.000)*	1.000 *(1.000-1.000)*	0.982 *(0.949-1.000)*	1.000 *(1.000-1.000)*	0.900 *(0.767-1.000)*
**Model 6**	0.904 *(0.671-1.000)*	0.818 *(0.544-0.993)*	1.000 *(1.000-1.000)*	0.786 *(0.583-1.000)*	0.987 *(0.987-1.000)*	0.926 *(0.833-0.984)*	0.989 *(0.933-1.000)*	0.776 *(0.544-1.000)*
**Model 7**	0.885 *(0.676-1.000)*	0.755 *(0.550-0.926)*	0.875 *(0.500-1.000)*	0.665 *(0.250-1.000)*	0.951 *(0.951-0.997)*	0.860 *(0.756-0.935)*	0.899 *(0.711-1.000)*	0.408 *(0.146-0.773)*
**Model 8**	0.904 *(0.671-1.000)*	0.881 *(0.600-1.000)*	1.000 *(1.000-1.000)*	0.806 *(0.615-1.000)*	0.997 *(0.997-1.000)*	0.970 *(0.925-0.998)*	0.989 *(0.989-1.000)*	0.789 *(0.517-1.000)*
**Model 9**	0.884 *(0.607-1.000)*	0.864 *(0.571-1.000)*	1.000 *(1.000-1.000)*	0.841 *(0.688-1.000)*	1.000 *(1.000-1.000)*	0.986 *(0.961-1.000)*	0.978 *(0.900-1.000)*	0.855 *(0.612-1.000)*
